# The beneficial application of preoperative 3D printing for surgical stabilization of rib fractures

**DOI:** 10.1371/journal.pone.0204652

**Published:** 2018-10-04

**Authors:** Ying-Yi Chen, Kuan-Hsun Lin, Hsu-Kai Huang, Hung Chang, Shih-Chun Lee, Tsai-Wang Huang

**Affiliations:** 1 Division of Thoracic Surgery, Department of Surgery, Tri-Service General Hospital, National Defense Medical Center, Taipei, Republic of China; 2 Graduate Institute of Medical Science, National Defense Medical Center, Taipei, Republic of China; University of Umeå, SWEDEN

## Abstract

**Objectives:**

The beneficial application of three-dimensional (3D) printing for surgical stabilization of rib fractures (SSRF) has never been proposed in the literature before. The aim of this study was to verify patients’ surgical outcomes when utilizing preoperative three-dimensional printing for SSRF.

**Methods:**

We retrospectively reviewed the records of all consecutive patients who were treated at our hospital for SSRF from July 2015 to December 2017. The patients were divided into two groups according to whether or not 3D printing was utilized.

**Results:**

Forty-eight patients who underwent SSRF at our hospital were enrolled. Of them, three patients underwent bilateral surgeries. The patients with application of preoperative 3D printing for SSRF had statistically significant associations with shorter operation time per fixed plate (*p* < 0.001), and a smaller incision length (*p* < 0.001).

**Conclusions:**

We present an useful technique involving 3D printing for promoting SSRF significantly with shorter operation time and an appropriate incision length.

## Introduction

Three-dimensional (3D) printing is a spectacular manufacturing technology with rapid prototyping, which enables the creation of 3D structures from computer-aided design data sets via an additive layering process. Until this report, there have been no definite conclusions in the literature about the clinical application of 3D printing in patients with rib fractures. Rib fractures are noted in 10% of all trauma patients and in about 30% of patients with significant chest trauma.[1] Surgical stabilization of rib fractures (SSRF) has traditionally required a big incision wound for adequate exposure; however, the degree of chest wall instability is determined by palpation, and the planned approach for surgery is fine-tuned on the basis of direct visualization of the rib fracture location during surgery. Recently, Schots et al.[2] mentioned that video-assisted thoracoscopic surgery (VATS) is effective and safe and can be of additional value by providing the possibility to adjust the planned incision for SSRF and to decrease the area of muscle destruction. However, the preoperative preparation of VATS is time-consuming, and single-lung ventilation and a prolonged operation time are expected. Moreover, at least one additional assistant is required to control the thoracoscopy process. We utilized 3D reconstruction from computed tomographic images to simulate the patient’s rib cage to determine the length and curve of the titanium plates before surgery to decrease the length of the incisions, identify the precise location of the fracture sites, and easily measure the rib thickness using a caliper to determine the proper screw length.

We have applied this useful technique to patients with rib fractures since January 2017. The aim of this study was to clarify the clinical benefits, in terms of surgical outcomes, of the application of 3D printing for SSRF.

## Materials and methods

### Patients who underwent surgical stabilization of rib fractures

From July 2015 to December 2017, we reviewed all cases of SSRF at our hospital according to clinical practice guidelines. Patients who received bilateral procedures were also included in the study. Patients who had lost of follow-up were excluded from the study. Follow-up information was obtained from the outpatient clinic medical reports. Emergent surgeries for rib fractures are the major cause for not available 3D printing. All patients with 3D printing SSRF received surgeries within 3 days. All adult patients who were included in our study gave informed consent with verbal agreement by themselves for medical research (not from parents or guardians). This study has been approved by the Institutional Review Board/Ethics Committee of Tri-Service General Hospital (1-106-05-078).

### Parameters

The medical records and operative notes of these patients were retrospectively reviewed. Data collected included detailed history, mechanism of chest trauma, sex, side, flail chest, age, body mass index in kg/m^2^, smoking habits, associated injuries, combined surgeries, surgical complications, injury severity score, the number of rib fractures, the number of fixed plates, total operation time, operation time per fixed rib, incision length, hospital stay (days), and intensive care unit (ICU) stay (days). Because surgical fixation for more rib fractures takes more operation time, we use operation time per fixed rib to express exactly the efficiency of surgery.

### Preoperative three-dimensional printing for surgical stabilization of rib fractures

**Fig 1:**
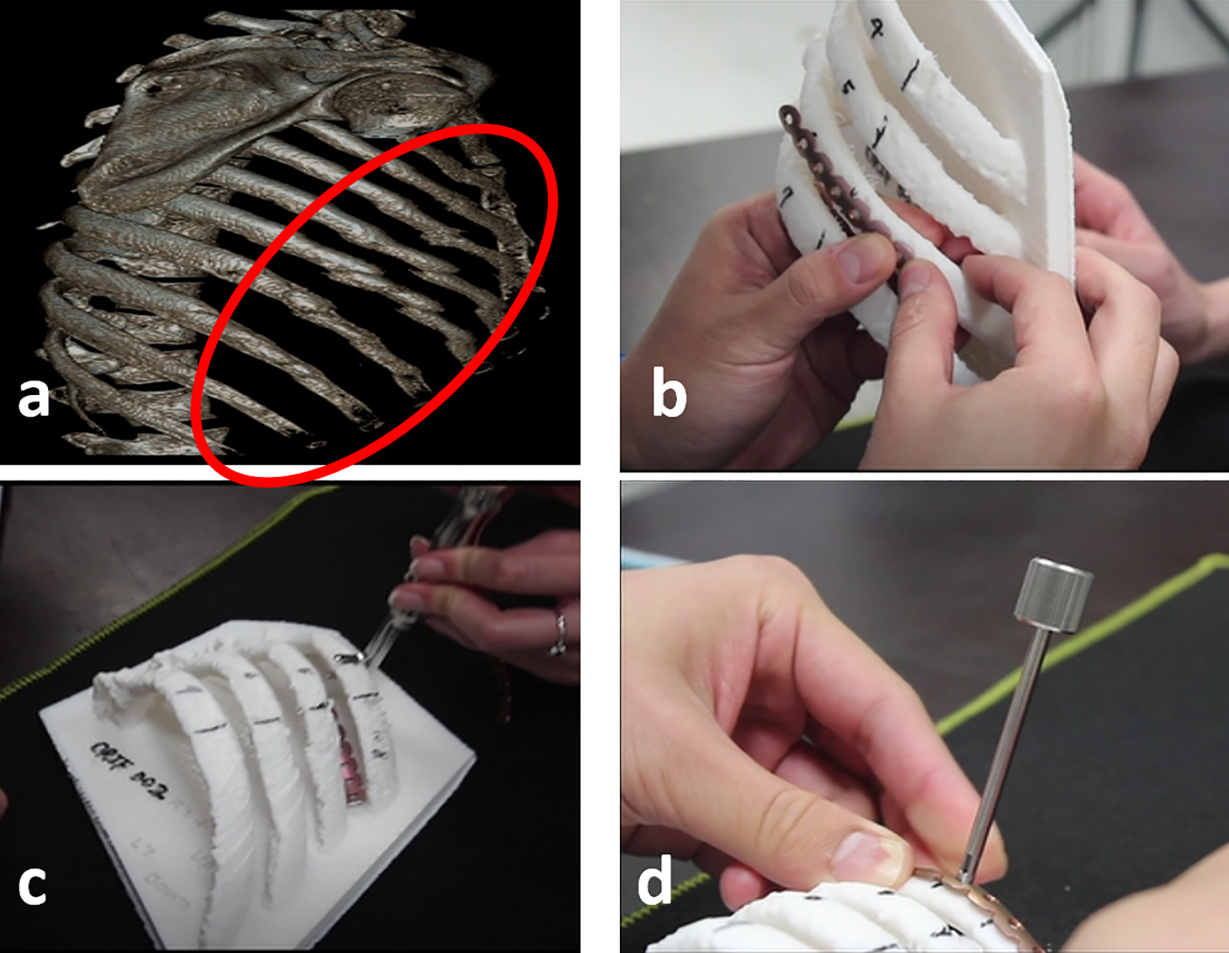
Illustrations of the preoperative designing process. **(a)** Reconstruction of chest computed tomography to generate preoperative three-dimensional image. **(b)** Measuring the length of titanium plate at least three holes on each side of rib fracture. **(c)** Using a calibre to measure the length of locking screws. **(d)** Bending the plates to fit the curvature.

**Fig 2:**
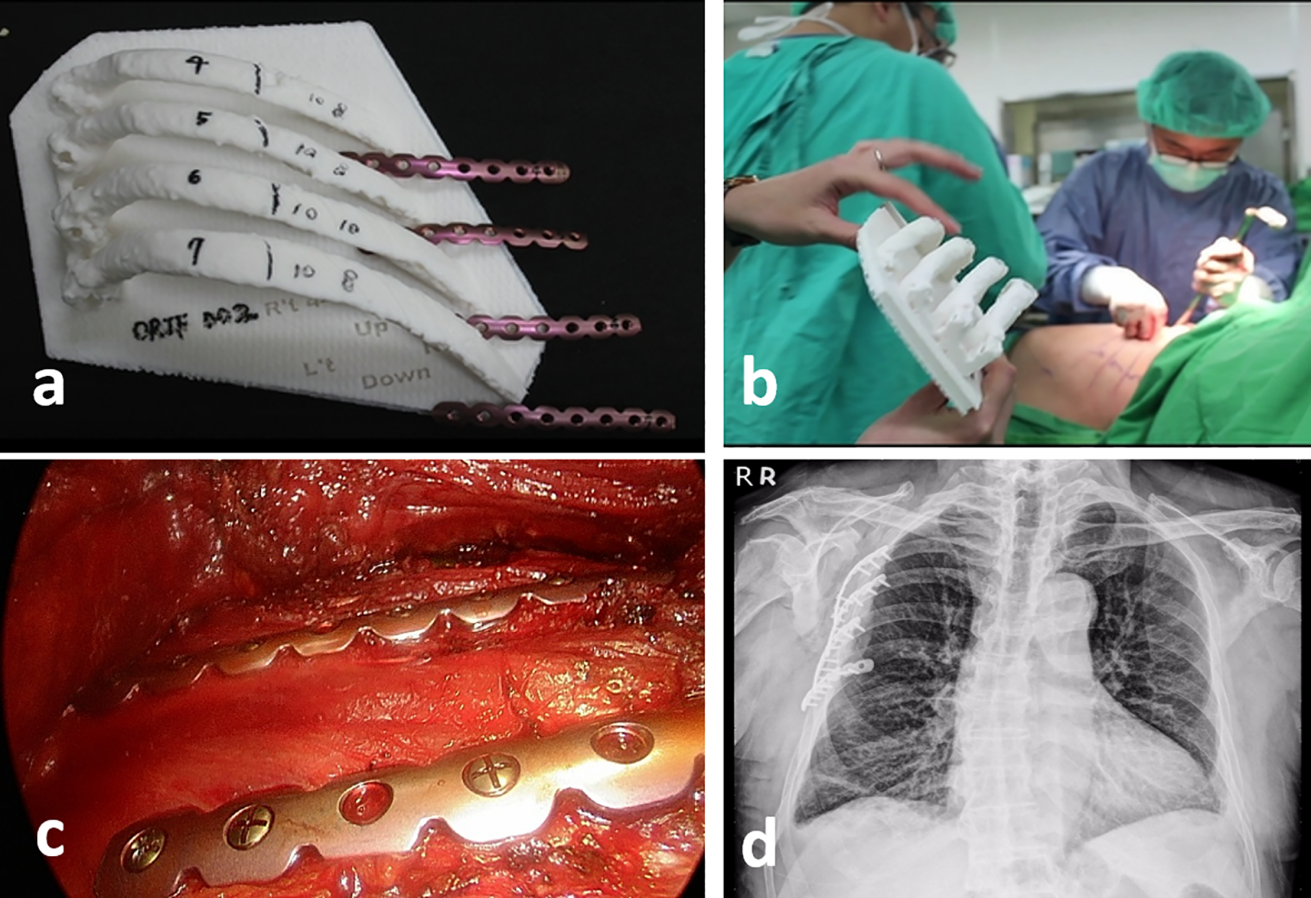
Illustrations of practical use in surgical stabilization of rib fractures. **(a)** Marking the annotations of the length of screws. **(b)** Utilizing the 3D printing model to localize the fracture sites. **(c)** Intraoperative view of fixed fractures. **(d)** The postoperative plain film of chest.

All patients with 3D printing assisted group received computed tomography (CT) of chest before surgery. 5 patients got CT of chest because of emergent conditions and others for the purpose of 3D printing. We utilized 1 mm slice thickness of CT image to create 3D CT reconstructions of the chest wall (Fig 1A) and then corrected the alignment of rib fractures. We used an UP-BOX 3D printer (Denford Ltd, UK master distributor) with acrylonitrile butadiene styrene as the raw material to produce a 3D-printed model of the ribcage. The MatrixRIB system (DePuy Synthes, Amersfoort, the Netherlands) provided anatomically contoured titanium angular stable plates for posterior and retroscapular fractures. The titanium plates were cut to a suitable length and were bent to fit the curve of the patient’s rib cage (Fig 1B). The rib thickness was measured using a caliper (Fig 1C). The plate was fixed with a minimum of three locking screws on each side of the fracture to ensure proper fixation (Fig 1D). After completion of the titanium plate designed for rib fixation (Fig 2A), the screws and titanium plates were sent for autoclave sterilization. The 3D printing-assisted rib fixation was performed with the patient under general anesthesia with standard single-lumen endotracheal intubation. Standard intravenous antibiotic prophylaxis was also administered. The patient was positioned in the lateral decubitus position with the use of a beanbag and without bending the table. According to the 3D-printed model, we made an incision along the fracture line of the ribs (Fig 2B). The skin incision was deepened through the subcutaneous tissue, and the latissimus dorsi and serratus anterior muscles were transected with electrocautery. The scapula was mobilized from the chest wall and retracted upward with a scapula retractor. After locating the rib fractures, any nonviable bone or debris from the fracture site was carefully cleaned away, and the fractured ribs were reduced with the use of reduction forceps that were inserted from the superior border of the rib initially. SSRF was performed in a cephalad-to-caudal and posterior-to-anterior fashion. The preoperatively designed plates were placed to the fractured rib and then temporarily secured with plate-holding forceps. We then used the selected screws (length decided by the 3D-printed model) to affix the titanium plates (Fig 2C). We also used a 90° drill and screwdriver for minimally invasive predrilling and insertion of screws without additional incisions. After full expansion of the lung, the procedure was terminated, and the incision was closed in layers in a routine manner with retention of one Jackson-Pratt drain. Postoperative chest plain film was routinely arranged (Fig 2D).

### Statistical analysis

Descriptive data were expressed as mean ± standard deviation, unless otherwise specified. The Student’s *t*-test (two-tailed t-test) was used to analyze continuous variables, whereas the chi-square test was used for comparison of categorical variables between the groups with and without 3D printing assistance for the SSRF. A P value of less than 0.05 was considered statistically significant. Statistical analyses were performed using SPSS version 22.0 software (IBM Corp., Armonk, NY, USA).

## Results

Forty-eight patients who received SSRF during the study period were included in the analysis, while five patients were excluded because of loss follow-up. In sixteen patients (33.3%) (17 surgeries), preoperative 3D printing was used to precontour plates for surgical reconstruction. Only three patients had bilateral procedures. In 28 of 48 patients, a motorcycle accident was the most common cause of the rib fractures. In 23 of 48 patients, a prominent hemothorax or lung penetrating injuries were present and needed proper evacuation or stapled wedge resection. Other bone fractures and brain injuries were the second most commonly associated injuries. In ten patients undergoing 3D printing with SSRF, we used VATS to explore the thoracic cavity and evacuate the hemothorax. Only one patient had traumatic aortic dissection, type B, and needed stenting of the descending aorta. All relevant mechanisms and associated injuries are listed in Table 1.

**Table 1:** Mechanisms, associated injuries, and combined surgeries in patients with surgical stabilization of rib fractures.

	SSRF (%) N = 32	3D for SSRF (%) N = 16
Mechanism:		
Fall	10 (31.25)	4 (25)
Motorcycle accident	20 (62.5)	8 (50)
Bicycle accident	1 (3.13)	0
Car accident	0	0
Pedestrian accident	1 (3.13)	4 (25)
Associated injuries:		
Brain	6 (18.75)	3 (18.75)
Lung	13 (40.63)	10 (62.5)
Liver	5 (15.63)	0
Spleen	3 (9.38)	0
Kidney	1 (3.13)	0
Great vessel	1 (3.13)	0
Other bone fractures	10 (31.25)	2 (12.5)
Combined surgery:		
VATS	13 (40.63)	10 (62.5)
ORIF (other bone)	8 (25)	2 (12.5)
Stenting to AD	1 (3.13)	0

Key: 3D, three-dimensional; SSRF, surgical stabilization of rib fractures; VATS, video-assisted thoracoscopic surgery; ORIF, open reduction with internal fixation; AD, aortic dissection

All relevant surgical treatment-related characteristics are listed in Table 2. There was no statistically significant difference in sex, side, smoking habit, flail chest, age, body mass index, injury-severity score, complications, number of rib fractures, or number of fixed plates between patients with and without 3D printing for SSRF. The mean operative time (175.24 vs 125 minutes, p = 0.003) and the mean operative time per fixed plate (52.99 vs 35.41 minutes, p < 0.001) was significantly shorter in patients with 3D printing for SSRF. The smaller incision length (14.19 vs 8.71 cm, p = 0.002) and wound length per fixed rib (4.19 vs 2.54 cm, p < 0.001) in patients with 3D printing for SSRF were statistically significant. Postoperatively, 30 patients were transferred to the ICU for close observation and monitoring. The mean duration of the ICU stay was not significantly different between groups. No patient required a tracheostomy. A routine chest roentgenogram was obtained on postoperative day 1. None of the roentgenograms led to an additional intervention. Only two patients who underwent SSRF had adverse postoperative events (one empyema and one wound infection), and they recovered with non-surgical management. There was no surgical mortality in this study. All patients were discharged from the hospital after 2 to 60 days, and the length of hospital stay was not significantly different between the two groups. The median follow-up time was 12 weeks (range, 4 to 40 weeks).

**Table 2:** Characteristics of patients with and without 3D printing application for surgical stabilization of rib fractures.

*Variables*	SSRF (%) n = 34	3D for SSRF (%) n = 17	*p-value*^a^
*Sex*			
*Male*	22 (68.75)	10 (62.5)	*0*.*673*
*Female*	10 (31.25)	6 (37.5)	
*Side*			
*Right*	14 (41.18)	10 (58.82)	*0*.*242*
*Left*	20 (58.82)	7 (41.18)	
*Flail chest*			
*Yes*	12 (35.29)	4 (23.53)	*0*.*403*
*No*	22 (64.71)	13 (76.47)	
*Smoking*			
*Yes*	16 (50)	5 (31.25)	*0*.*434*
*No*	16 (50)	11 (68.75)	
*Age (year)*	51.44 ± 14.59	54.29 ± 14.43	*0*.*512*
*BMI (kg/m2)*	24.77 ± 3.21	25.04 ± 3.48	*0*.*413*
*ISS*	22.74 ± 8.74	21 ± 8.94	*0*.*510*
*Number of rib fractures*	5.91 ± 1.88	5.18 ± 1.78	*0*.*186*
*Number of fractures repaired*	3.47 ± 1.35	3.59 ± 0.87	*0*.*746*
*Operation time (minute)*	175.24 ± 60.58	125 ± 33.44	*0*.*003*^b^
*OP time per fixed rib (minute)*	52.99 ± 14.93	35.41 ± 8.58	*< 0*.*001*^b^
*Wound length (cm)*	14.19 ± 6.86	8.71 ± 2.11	*0*.*002*^b^
*Wound length per fixed rib (cm)*	4.19 ± 1.52	2.54 ± 0.67	*< 0*.*001*^b^
*Hospital stay (day)*	20.88 ± 12.73	18.94 ± 14.49	*0*.*626*
*ICU stay (day)*	11.9 ± 8.09	6.44 ± 5.53	*0*.*076*
*Complications*			
*Broken plates*	2 (5.88)	0	*0*.*215*
*Empyema*	1 (2.94)	0	
*Wound infection*	1 (2.94)	0	

^a^Significance was assessed using χ^2^ tests.

^b^Significance was assessed using Student’s *t* tests.

Key: BMI, body mass index; ISS, injury severity score; OP, operation; ICU, intensive care unit

## Discussion

3D printing has been developed in many fields of biomedicine. Vivek Baskaran [3] investigated the applications of 3D printing for anatomy and surgical education and neurosurgery in the literature review of the PubMed and Web of Science databases. These applications significantly improved the quality of anatomy and surgical education and practices. 3D printing produced accurate simulations of patient-specific anatomy, which can then be used for preoperative planning and skill acquisition and demonstrates advantages in cadaveric dissection, plastic models, and plastination of cadaver specimens [4]. We propose that our data is the first retrospective cohort study to identify the efficacy and benefits of 3D printing for SSRF.

Over 30% of patients with severe chest trauma have some long-term disability and often do not return to full-time employment. [5] Additionally, chest trauma results in social and economic costs, both to the U.S. healthcare system and to individuals, lost productivity, and decreased quality of life. [6] Bhatnagar [7] presented a model that looked specifically at long-term outcomes and quality of life after SSRF in patients treated in North American trauma centers, and this Markov decision model was designed to compare the cost-effectiveness of nonoperative management versus SSRF of a flail chest. Numerous studies examining the surgical repair of ribs promote this technique as superior to conservative treatment. [8–12] Therefore, in order to ensure a minimally invasive approach, Nickerson [13] mentioned that the surgical reach for rib fracture stabilization can be extended with the use of a 90° drill and screwdriver and that high fractures under the scapula where access is technically challenging can be stabilized without prolonged operative times. However, there is still much room for improvement of the surgical process, such as shortening the incision, reducing the time spent on searching for fracture sites, designing the length and curvature of the titanium plates, and measuring the length of the locking screws.

We utilized 3D printing to create a personalized design model to assist in rib fixation surgery. As shown in Table 2, our data showed that 3D printing for SSRF significantly reduced the operation time and aided in preoperatively determining the surgical plans, such as the location and length of the incision. Therefore, the preoperative 3D printing approach has the following three advantages for SSRF: (1) Ease of locating the rib fracture site and predicting the incision length, (2) Shortened operation time, (3) Useful for explaining the steps of SSRF to patients and families. In our patients, we only did SSRF among 3^rd^-10^th^ ribs. Not all rib fractures should be fixed, because stabilization of chest wall is the most important. Thus, 1^st^-2^nd^ and 11^th^-12^th^ rib fractures might not be fixed, except for associated great vessel injuries or other organ injuries. Actually, time consuming of intraoperative customization of plates depends on how much fragments of ribs and measuring thickness and curvature of different sites of ribs.

However, our approach has some limitations in SSRF. If a patient has other cardiothoracic injuries, thoracotomy with extended exploration might be necessary to check bleeding. Thus, a 3D printed model for SSRF would be superfluous. This new technique is not suitable for emergency conditions because 3D printing is time-consuming (at least 5–6 hours). We used computed tomographic images to generate 3D printing model in all kinds of rib fractures, even in flail of chest or non-aligned rib fractures. The cost is an additional limitation. Rapid prototyping machines cost $100 to $200 without including the cost of the plastic and resin-based materials. The developments of 3D printing will include different range of materials and lead to more durable and realistic products in the future by the advancement of technology. Berman [14] proposed that the cost and speed of 3D printers will improve, thereby increasing the usability and potentially the availability of these machines. Liman [3] said 3D printing will potentially revolutionize the anatomical and surgical sciences to the benefit of educators, surgeons, and patients in the future. Therefore, the 3D printing technique could become more valuable for rib fixation surgery. Prospective multi-institutional studies are needed to validate the feasibility and safety of 3D printing for the SSRF. Further clinical studies may provide more valuable results for patient outcomes.

## Conclusion

3D printing for SSRF is a safe and practical technique because of no complications compared with SSRF. We also proved that it resulted in a significantly shorter operation time and an appropriate incision length.

## References

[1] Liman S. Chest injury due to blunt trauma. European Journal of Cardio-Thoracic Surgery. 2003;23(3):374–8. 10.1016/s1010-7940(02)00813-8 12614809

[2] Schots JP, Vissers YL, Hulsewe KW, Meesters B, Hustinx PA, Pijnenburg A, et al Addition of Video-Assisted Thoracoscopic Surgery to the Treatment of Flail Chest. Ann Thorac Surg. 2017;103(3):940–4. 10.1016/j.athoracsur.2016.09.036 . 27939010

[3] Baskaran V, Strkalj G, Strkalj M, Di Ieva A. Current Applications and Future Perspectives of the Use of 3D Printing in Anatomical Training and Neurosurgery. Front Neuroanat. 2016;10:69 10.3389/fnana.2016.00069 ; PubMed Central PMCID: PMCPMC4919320. 27445707 PMC4919320

[4] McMenamin PG, Quayle MR, McHenry CR, Adams JW. The production of anatomical teaching resources using three-dimensional (3D) printing technology. Anat Sci Educ. 2014;7(6):479–86. 10.1002/ase.1475 . 24976019

[5] Beal SL, Oreskovich MR. Long-term disability associated with flail chest injury. Am J Surg. 1985;150(3):324–6. . 4037191 10.1016/0002-9610(85)90071-6

[6] Majercik S, Cannon Q, Granger SR, VanBoerum DH, White TW. Long-term patient outcomes after surgical stabilization of rib fractures. Am J Surg. 2014;208(1):88–92. 10.1016/j.amjsurg.2013.08.051 . 24507379

[7] Bhatnagar A MJ, Nirula R. Rib fracture fixation for flail chest: what is the benefit? JACS. 2012;215:201–5. 10.1016/j.jamcollsurg.2012.02.023 22560319

[8] Tanaka H, Yukioka T, Yamaguti Y, Shimizu S, Goto H, Matsuda H, et al Surgical stabilization of internal pneumatic stabilization? A prospective randomized study of management of severe flail chest patients. J Trauma. 2002;52(4):727–32; discussion 32. . 11956391 10.1097/00005373-200204000-00020

[9] Marasco SF, Davies AR, Cooper J, Varma D, Bennett V, Nevill R, et al Prospective randomized controlled trial of operative rib fixation in traumatic flail chest. J Am Coll Surg. 2013;216(5):924–32. 10.1016/j.jamcollsurg.2012.12.024 . 23415550

[10] Nirula R, Allen B, Layman R, Falimirski ME, Somberg LB. Rib fracture stabilization in patients sustaining blunt chest injury. Am Surg. 2006;72(4):307–9. . 16676852 10.1177/000313480607200405

[11] Tarng YW, Liu YY, Huang FD, Lin HL, Wu TC, Chou YP. The surgical stabilization of multiple rib fractures using titanium elastic nail in blunt chest trauma with acute respiratory failure. Surg Endosc. 2016;30(1):388–95. 10.1007/s00464-015-4207-9 ; PubMed Central PMCID: PMCPMC4710669. 25875089 PMC4710669

[12] Althausen PL, Shannon S, Watts C, Thomas K, Bain MA, Coll D, et al Early surgical stabilization of flail chest with locked plate fixation. J Orthop Trauma. 2011;25(11):641–7. 10.1097/BOT.0b013e318234d479 . 22008858

[13] Nickerson TP, Kim BD, Zielinski MD, Jenkins D, Schiller HJ. Use of a 90 degrees drill and screwdriver for rib fracture stabilization. World J Surg. 2015;39(3):789–93. 10.1007/s00268-014-2862-y ; PubMed Central PMCID: PMCPMC4882093. 25403887 PMC4882093

[14] Berman B. 3-D printing: The new industrial revolution. Business Horizons. 2012;55(2):155–62. 10.1016/j.bushor.2011.11.003

